# Supplementary material for: Mechanotransductive Activation of PPAR-γ by Low-Intensity Pulsed Ultrasound Induces Contractile Phenotype in Corpus Spongiosum Smooth Muscle Cells
Source: Biomedicines. 2025 Jul 11;13(7):1701. doi: 10.3390/biomedicines13071701 (PMC12292440; doi:10.3390/biomedicines13071701)
Supplement: Supplementary file 1 [file biomedicines-13-01701-s001.zip › biomedicines-3697442-supplementary.pdf]

**Supplemental Table S1: Primers Used for RT-qPCR Analysis**

| **Gene** | **Sequence** | |
|---|---|---|
| **Human Primers** | | |
| **α-SMA** | Forward | 5’-AGGTAACGAGTCAGAGCTTTGGC-3’ |
| | Reverse | 5’-CTCTCTGTCCACCTTCCAGCAG-3’ |
| **SM-22α** | Forward | 5’-CTGTCAGCCGAGGTTAAGAAC-3’ |
| | Reverse | 5’-GAGGCCGTCCATGAAGTTGTT-3’ |
| **Collagen I** | Forward | 5’-CTTCCTACGGGGAATCTGTGT-3’ |
| | Reverse | 5’-CAATGGCGTTTTGGGTGTTC-3’ |
| **OPN** | Forward | 5’-TGAAATTCATGGCTATGGAA-3’ |
| | Reverse | 5’-TGAAACGAGTCAGCTGGATG-3’ |
| **PPAR γ** | Forward | 5’-TGAATCCAGAGTCCGCTGACCTC -3’ |
| | Reverse | 5’-ATCGCCCTCGCCTTTGCTTTG-3’ |
| **GAPDH** | Forward | 5’-ACAACTTTGGTATCGTGGAAGG-3’ |
| | Reverse | 5’-GCCATCACGCCACAGTTTC-3’ |
